# Supplementary material for: Changes in the Volatile Flavor Substances, the Non-Volatile Components, and the Antioxidant Activity of Poria cocos during Different Drying Processes
Source: Molecules. 2024 Oct 9;29(19):4777. doi: 10.3390/molecules29194777 (PMC11478257; doi:10.3390/molecules29194777)
Supplement: Supplementary file 1 [file molecules-29-04777-s001.zip › molecules-3221032-supplementary.pdf]

Table S1 Volatile flavor compounds content and their contents in the *Poria cocos* samples during drying

Code number	Name of compounds	Odor descriptor	Content/%							
			HD50	HD60	HD70	HD80	HD90	HD100	SD	ID
Aldehydes										
1	Hexanal	Green, refreshing, fatty	6.61±0.25^{c}	9.01±0.42^{ab}	9.05±0.07^{ab}	10.04±0.42^{a}	9.87±0.18^{a}	7.97±0.06^{bc}	2.23±0.82^{d}	7.14±0.14^{c}
2	(E)-2-Hepten-1-al	green	-	0.91±0.04^{c}	1.54±0.21^{abc}	0.98±0.04^{bc}	1.62±0.31^{ab}	2.24±0.05^{a}	1.25±0.16^{bc}	1.54±0.30^{abc}
3	2-Octenal, (E)-	Fatty, green, nutty	1.35±0.10^{c}	2.84±0.34^{ab}	3.26±0.75^{ab}	3.78±0.29^{ab}	3.91±0.19^{a}	3.48±0.53^{a}	2.31±0.26^{b}	2.51±0.05^{abc}
4	Nonanal	Sweet, floral, rose	3.73±0.20^{bc}	4.55±0.37^{abc}	4.91±0.52^{ab}	3.38±0.23^{c}	3.76±0.16^{bc}	4.01±0.37^{abc}	5.07±0.44^{a}	4.50±0.16^{abc}
5	2-Nonenal, (E)-	Bean, grass, fatty	2.28±0.03^{ab}	3.42±0.37^{a}	2.68±0.37^{ab}	1.93±0.49^{b}	2.84±0.10^{ab}	2.54±0.34^{ab}	3.03±0.06^{a}	3.19±0.10^{a}
6	Decanal	Sweet, fatty, orange	1.93±0.22^{a}	1.47±0.08^{abc}	1.81±0.42^{abc}	1.41±0.08^{abc}	1.07±0.03^{bc}	1.02±0.03^{c}	1.96±0.16^{a}	1.90±0.32^{ab}
7	Undecanal	Citrus, fatty, sweet	0.95±0.01^{a}	1.75±0.24^{a}	0.63±0.26^{a}	0.96±0.19^{a}	-	-	0.80±0.05^{a}	1.13±0.84^{a}

8	Dodecanal	Citrus, fruity, sweet	-	-	-	-	-	-	0.94±0.22	-
9	Caryophyllene	woody	13.29±0.11^{ab}	12.19±0.44^{ab}	11.41±0.50^{b}	14.01±1.07^{a}	13.33±0.37^{ab}	11.75±1.00^{ab}	6.24±0.49^{c}	12.68±2.11^{ab}
subtotal			30.14±0.92^{ab}	35.96±2.30^{a}	35.28±3.10^{a}	34.49±2.82^{a}	36.39±1.34^{a}	33.01±2.36^{a}	23.82±2.86^{b}	34.59±2.11^{a}
Hydrocarbons										
10	β-Myrcene	Pungent, woody	4.57±0.31^{c}	6.65±0.23^{b}	7.22±0.57^{b}	6.61±0.25^{b}	8.17±0.27^{b}	10.49±1.19^{a}	-	6.43±0.05^{bc}
11	D-Limonene	Orange	-	-	1.21±0.21^{c}	1.33±0.06^{c}	1.78±0.13^{a}	1.79±0.17^{a}	0.98±0.06^{c}	1.10±0.06^{c}
12	Bicyclo [3.1.0] hexane, 4-methylene-1-(1-methylethyl)-	woody	2.39±0.33^{a}	-	1.88±0.26^{a}	2.28±0.13^{a}	1.88±0.05^{a}	2.53±0.13^{a}	1.15±0.12^{b}	1.75±0.63^{a}
13	3,5-Octadien-2-ol	-	-	-	-	-	2.09±0.51^{a}	-	-	0.95±0.20^{b}
14	Dodecane	-	-	0.70±0.25^{a}	0.76±0.21^{a}	0.85±0.25^{a}	1.02±0.14^{a}	0.97±0.18^{a}	-	-

15	Tridecane	Alkane	0.73±0.18a	0.96±0.18a	1.20±0.27a	0.91±0.01a	1.18±0.16a	1.06±0.35a	0.56±0.02a	0.91±0.24a
16	Cyclohexane, 1-ethenyl-1-methyl-2,4-bis(1-methylethenyl)-, [1S-(1.alpha.,2β,4β)]-	sweet	1.80±0.14cd	1.25±0.19d	2.52±0.29ab	2.99±0.05a	2.17±0.18bc	1.80±0.01cd	1.29±0.16d	1.77±0.01cd
17	Tetradecane	Alkane	2.08±0.23b	3.02±0.03a	2.90±0.39ab	2.74±0.01ab	3.23±0.16a	2.93±0.45ab	1.14±0.03c	2.55±0.01ab
18	Cyclohexane, 1-ethenyl-1-methyl-2-(1-methylethenyl)-4-(1-methylethylidene)-	-	-	-	0.97±0.44a	0.97±0.10a	-	-	0.60±0.15a	-
19	(E)-β-Famesene	woody	1.57±0. 19a	1.78±0.70a	1.60±0.22a	1.63±0. 19a	1.40±0.84a	1.42±0.50a	0.98±0.05a	0.88±0. 11a
20	β-Eudesmene	woody	1.67±0. 18a	2.29±0.06a	1.59±0.05a	1.47±0.82a	1.16±0. 11a	1.69±0.28a	-	1.71±0.07a
21	4a,8-Dimethyl-2-(prop-1-en-2-yl)-	herbal	1.10±0.24ab	1.59±0.24a	0.95±0.03b	1.50±0.15ab	1.59±0.21a	1.38±0.04ab	-	1.17±0.28ab

	1,2,3,4,4a,5,6,7-octahydronaphthalene									
22	Naphthalene, 1,2,3,5,6,8a-hexahydro-4,7-dimethyl-1-(1-methylethyl)-, (1S-cis)-	woody	1.27±0.06a	1.04±0.08a	0.93±0.08a	-	1.07±0.16a	0.99±0.12a	-	
23	Pentadecane, 3-methyl-	Alkane	2.09±0.04a	2.29±0.35a	2.41±0.54a	2.03±0.02ab	2.03±0.17ab	1.83±0.02ab	1.26±0.06b	2.14±0.06a
24	Caryophyllene oxide	Sweet, fresh, woody	-	-	-	1.58±0.07a	1.51±0.02a	1.46±0.06a	1.11±0.35a	-
25	Hexadecane	Alkane	1.49±0.01a	1.48±0.26a	1.61±0.19a	1.73±0.22a	1.54±0.11a	1.45±0.12a	1.27±0.21a	1.75±0.08a
subtotal			20.54±2.18c	23.04±2.57b	27.73±3.72ab	28.61±2.33a	31.82±3.62a	31.80±3.62a	10.32±1.08d	23.12±1.79bc
Alcohols										
26	2-Ethyl-1-hexanol	Special	1.71±0.03a	1.93±0.05a	1.95±0.48a	1.87±0.41a	1.91±0.01a	1.49±0.05ab	0.89±0.08b	1.99±0.27a
27	Linalool	Floral, sweet, fruity	1.87±	1.87±	1.61±	2.23±	2.42±	1.38±	1.61±	2.24±

			0.10_{ab} 0.10^{b}	0.04_{ab} 0.22_{a}	0.08^{b}		0.35_{a}	0.45_{a}	0.37^{b}	
28	1-Nonanol	floral	-	-	-	-	-	-	1.42± 0.12	-
29	1-Hexadecanol	waxy	-	1.78± 0.15_{a}	1.60± 0.22_{ab}	-	0.96± 0.05^{b}	1.21± 0.52_{ab}	-	0.73± 0.01^{b}
30	1,6,10-Dodecatrien-3-ol, 3,7,11-trimethyl-, (E)-	Floral, citrus, woody	4.89± 0.33^{b}	2.82± 0.05_{c}	2.45± 0.48_{c}	3.29± 0.76_{c}	-	-	7.34± 0.38_{a}	3.36± 0.29_{c}
31	Cyclopropanemethanol, α.,2-dimethyl-2-(4-methyl-3-pentenyl)-, [1. α.(R*),2. α.]-	-	-	-	-	-	-	-	1.46± 0.05	-
subtotal			8.47± 0.47^{b}	8.40± 0.28^{b}	7.61± 1.27^{b}	7.49± 1.51^{b}	5.29± 0.46_{c}	4.08± 0.94_{c}	12.73± 0.72_{a}	8.32± 0.78^{b}
Esters and Ketones										
32	Butyric acid, 2,2-dimethyl-, vinyl ester	-	0.71±	2.15±	2.33±	1.82±	2.63±	1.85±	1.23±	1.79±
				0.19^{d}		0.53_{ab}		0.34_{ab}	0.03^{bc}	0.15_{a}

0.04^{b} 0.01_{cd} 0.12^{bc}

33	Nonanoic acid	waxy	-	-	--	-	-	0.62±0.01^{b}	1.59±0.05$_{a}$	-
34	2,2,4-Trimethyl-1,3-pentanediol diisobutyrate	-	0.46±0.03$_{c}$	0.68±0.19^{bc}	-	0.60±0.16^{bc}	0.59±0.22$_{c}$	1.19±0.10$_{a}$	1.02±0.09$_{ab}$	-
35	Butanoic acid, butyl ester	fruity	-	-	-	-	-	-	4.33±0.57$_{a}$	1.83±0.05^{b}
36	Nonanoic acid, 9-oxo-, methyl ester	-	-	-	-	-	-	-	0.44±0.00	-
37	Isopentyl 3,5,5-trimethylhexanoate	-	6.04±1.48$_{a}$	2.21±0.35^{b}	1.66±0.57^{b}	2.24±0.28^{b}	0.98±0.02^{b}	0.54±0.35^{b}	2.27±0.23^{b}	1.93±0.45^{b}
38	Pentadecanoic acid, methyl ester	-	2.44±0.08^{b}	1.10±0.21^{c}	0.98±0.18$_{c}$	1.12±0.04$_{c}$	-	-	3.31±0.26$_{a}$	1.46±0.14$_{c}$
39	1,2-Benzenedicarboxylic acid, bis(2-methylpropyl) ester	-	-	-	-	-	-	-	1.21±0.08	-
40	Hexadecanoic acid,	waxy	3.75±	1.48±	1.67±	1.72±	-	-	4.81±	1.83±

	methyl ester		0.48a	0.35b	0.42b	0.52b			0.26a	0.67b
41	Methyl linoleate	Fatty, woody	4.70±0.28a	1.35±0.21bcd	2.28±0.25bc	2.02±0.38bc	0.56±0.01cd	0.12±0.01d	5.05±0.06a	2.47±0.70b
42	2-Undecanone	Fatty, fruity	1.07±0.29b	1.50±0.29ab	1.74±0.05ab	1.69±0.29ab	1.67±0.03ab	1.60±0.20ab	2.09±0.02a	2.13±0.18a
43	5,9-Undecadien-2-one, 6, 10-dimethyl-	floral	1.48±0.11a	1.53±0.82a	1.35±0.03a	1.25±0.15a	0.85±0.08a	1.26±0.06a	1.32±0.23a	1.49±0.06a
subtotal			20.64±2.94b	12.01±2.49cd	12.01±2.61cd	12.47±1.84cd	7.28±0.41d	7.17±0.71d	28.66±1.82a	14.94±2.36bc
others										
44	Benzene, [[(1-ethenyl-1,5-dimethyl-4-hexenyl) oxy] methyl]-	-	2.92±0.20ef	4.48±0.37c	5.09±0.21c	4.24±0.02cd	6.07±0.36b	9.48±0.41a	2.14±0.05f	3.35±0.23de
45	(E)-Sesquisabinene hydrate	-	1.25±0.15ab	1.14±0.19b	1.09±0.19b	1.43±0.09ab	1.67±0.07a	1.37±0.03ab	-	1.52±0.18ab
46	α-Selinene	-	3.79±0.26abc	3.12±0.11cd	3.75±0.14a	4.03±0.06ab	4.43±0.11a	4.03±0.15ab	2.14±0.34d	3.67±0.23bc
47	.gamma.-Muurolene	-	0.73±	4.25±	0.53±	1.37±	0.63±	0.60±	-	1.14±

	0.02$_{cd}$	0.21^{a}	0.05^{d}	0.24^{b}	0.16^{d}	0. 11^{d}		0.03^{bc}
subtotal	8.68±0.63$_{e}$	13.35±0.88^{b}	10.46±0.60$_{cd}$	11.06±0.40$_{c}$	12.81±0.70^{b}	15.49±0.69$_{a}$	4.28±0.39^{f}	9.68±0.67^{de}

Note: "-" indicates that is not detected or below detection limit. Data points in the same row with different letters are significantly different ($p < 0.05$). HD means hot air drying, SD means integrated shade-drying, ID means conventional infrared drying.
